# Time for a standardized system of reporting sites of genomic methylation

**DOI:** 10.1186/s13059-015-0654-9

**Published:** 2015-04-30

**Authors:** Richard Saffery, Lavinia Gordon

**Affiliations:** Cancer & Disease Epigenetics, Murdoch Childrens Research Institute, Royal Children’s Hospital, Flemington Road, Parkville, Australia; Department of Paediatrics, University of Melbourne, Parkville, VIC 3010 Australia; Australian Genome Research Facility Ltd, Royal Parade, Parkville, VIC 3050 Australia

## Abstract

The authors argue that the lack of a widely used, systematic way to report sites of DNA methylation is a often a barrier to reproducibility and therefore holds back research.

The analysis of DNA methylation has become commonplace in research and many thousands of studies that rely on this technique have been reported. Nevertheless, comparisons between these studies and the replication of their findings, which are key to gaining a true understanding of the role differential methylation in health and disease, are routinely hampered by the lack of consistency in reporting sites of differential methylation. In the absence of a common and systematic approach to naming such sites, attempts at reproducing previously reported findings are often time consuming or even impossible. Efforts are hindered by inappropriate (arbitrary) naming of sites of methylation and/or by a lack of reporting of the necessary information needed to replicate data faithfully.

At present, it is often necessary to search through pages of supplementary data in an attempt to identify assay details; these often take the form of amplification primer sequences that are designed for bisulfite-converted DNA, which shows little resemblance to the unconverted genome sequence being interrogated. A commonly used approach for reporting is to refer to a nearby landmark, such as the transcriptional start site (TSS) of a neighboring gene (for example, ‘-45 from the TSS’). However, this is of limited utility for the majority of genomic CpG sites, which are found in regions located far from such landmark sites. The situation is further complicated by the newly appreciated abundance of alternative TSSs for many genes.

In order to facilitate the rapid replication of methylation data, we propose a universal system for identifying specific sites of DNA methylation. There are two ways that this could be done. First, in accordance with approaches for single nucleotide polymorphism (SNP) identification, it could be achieved through the sequential numbering of sites as they are identified. This has led to the ‘rs’ nomenclature and the many tens of millions of sequence variants that are currently represented in databases such as dbSNP (www.ncbi.nlm.nih.gov/SNP/), each with a unique identifier. Although this is a practical system, newly deposited variants are now being identified by a number of up to nine digits (for example, rs1457689123) that provides no information on genomic context or type of sequence variant. This information needs to be sourced independently. A corresponding approach for CpG sites in the human genome would be anticipated to have more than 28 million identifiers for each unique CpG site, as well as many more identifiers for non-CpG sites of methylation. To some extent, this approach has already been adopted in the form of the reporting of the defined names of probes that are found on commonly used array platforms for methylation profiling (for example, cg12345678 of the Illumina Infinium HumanMethylation bead arrays). However, these probes represent only a small proportion of potential methylation sites, are limited to the human genome, and often cover more than a single site of potential methylation. Thus, a naming system based on this platform is of limited utility.

An alternative approach would take advantage of the currently annotated genomic location of specific sites as part of the naming system. This has the advantage of providing immediate spatial context to the associated site, in addition to providing a unique identifier for each location. The type of methylation site (CpG, CpH; H=A,C,T) can be included in the identifier, along with chromosome location. A caveat is that such an identifier only has utility when the reference genome build is also provided. Accordingly, a site on chromosome 1 that is labeled CpG1:3435353 has a potentially different location in the latest human genome build (hg19) to that in hg18 (the previous build, which is still widely used). Nevertheless, we favor this approach of identifying methylation sites as it is already widely in use for describing the location of many other specific genomic features, so there should be no barrier to adopting such a system for reporting sites of DNA methylation. This approach also has the major advantage that researchers can immediately identify the genomic location of any reported methylation findings.

The time has come to adopt a uniform approach to the identification of specific sites of genomic methylation in order to facilitate the replication of studies. We encourage comment and discussion from other researchers in the epigenomics community, including the editors of journals that routinely publish DNA methylation data. We also suggest that researchers carrying out methylation analyses should consider reporting sites of methylation using this system. As a first step towards this, we have generated a dedicated CpG bed file with ID numbers for all sites in the human hg19 genome assembly for use in the UCSC Genome Browser (http://genome.ucsc.edu/). This is available from us on request. We anticipate the rapid availability of similar resources for other genome builds, including those of non-human species.

## Abbreviation

TSS: Transcriptional start site

